# Zero-Valent Iron Nanoparticles for Soil and Groundwater Remediation

**DOI:** 10.3390/ijerph17165817

**Published:** 2020-08-11

**Authors:** Alazne Galdames, Leire Ruiz-Rubio, Maider Orueta, Miguel Sánchez-Arzalluz, José Luis Vilas-Vilela

**Affiliations:** 1 Macromolecular Chemistry Group (LQM), Physical Chemistry Department, Faculty of Science and Technology, University of the Basque Country, 48940 Leioa, Spain; alazne.galdames@ehu.eus (A.G.); joseluis.vilas@ehu.eus (J.L.V.-V.); 2 BCMaterials, Basque Center for Materials, Applications and Nanostructures, UPV/EHU Science Park, 48940 Leioa, Spain; 3 Iragaz Watin S.A., 20720 Azkoitia, Spain; morueta@iragaz.com (M.O.); msanchez@iragaz.com (M.S.-A.)

**Keywords:** soil remediation, nanoparticles, NZVI, zero-valent iron, bimetallic nanoparticles, stabilized zero-valent iron nanoparticles, pilot-scale

## Abstract

Zero-valent iron has been reported as a successful remediation agent for environmental issues, being extensively used in soil and groundwater remediation. The use of zero-valent nanoparticles have been arisen as a highly effective method due to the high specific surface area of zero-valent nanoparticles. Then, the development of nanosized materials in general, and the improvement of the properties of the nano-iron in particular, has facilitated their application in remediation technologies. As the result, highly efficient and versatile nanomaterials have been obtained. Among the possible nanoparticle systems, the reactivity and availability of zero-valent iron nanoparticles (NZVI) have achieved very interesting and promising results make them particularly attractive for the remediation of subsurface contaminants. In fact, a large number of laboratory and pilot studies have reported the high effectiveness of these NZVI-based technologies for the remediation of groundwater and contaminated soils. Although the results are often based on a limited contaminant target, there is a large gap between the amount of contaminants tested with NZVI at the laboratory level and those remediated at the pilot and field level. In this review, the main zero-valent iron nanoparticles and their remediation capacity are summarized, in addition to the pilot and land scale studies reported until date for each kind of nanomaterials.

## 1. Introduction

Soil pollution is an arising concern worldwide; it could be defined as the presence of contaminants, persistent toxic compounds, and hazardous substances, in soil. These pollutants must be present in the soil in a concentration beyond a threshold limit, being this limit the concentration beyond which can be injurious or harmful for human and animal health and plant growth [1]. Soil contamination can be caused due to several factors like improper management of urban and industrial waste, chemical spillage, commonly, due to industrial activity, and excessive usage of fertilizers and pesticides in agriculture [2].

Faced with the unconcern and uncontrolled spills of past times, in recent decades soil pollution has been raised as a serious concern due to the great importance of preserving soil quality for ecosystems and human health. The most widely used remediation methods are usually based on two main methodologies, in situ or ex situ remediation [3,4]. Both in situ and on site remediation could be performed by different methods such as solidification and stabilization, oxidation, soil vapor extraction, bioremediation, or nanoremediation [4,5,6,7]. On one hand, in situ soil treatment is a method in which the contaminated soil is treated without removing it. This method is especially interesting because it minimizes the alteration of characteristics such as soil structure and integrity [8,9]. However, this method, frequently, presents a lower remediation potential, being often considered time-consuming and presenting many uncertainties during the process. Also, considering the potential risks, environment and/or human health, this technique could not be suitable for its application at certain sites [10].

On the other hand, on site method required of the excavation of contaminated soil before its treatment, and placed in adequate container or tanks where the treatment will be carried out. After the treatment, the soil will be replaced to its original site [4,11].

However, due to the complex nature of most contaminated soils and the fact that contamination is often caused by the presence of a mixture of contaminants, the application of more than one remediation technique is, in many cases, required to reduce the concentration of contaminants to acceptable levels [12].

In this work, the recent advances on use of zero-valent iron nanoparticles-based technologies for soil and groundwater remediation are reviewed. The main types of zero-valent iron nanoparticles used in nanoremediation have been described. In addition, an especial attention has been made in the review of those studies carried out at pilot or full scale for all described nanoparticulate system.

## 2. Zero-Valent Iron

The use of zero-valent metals for environmental applications was first described in 1972 [13]. Years later, the degradation of trichloroethylene (TCE) in the presence of several metals, mainly zero-valent iron (ZVI), was demonstrated. This was considered the starting point of numerous subsequent studies in this area, beginning with the use of zero-valent metals for the remediation of groundwater contaminated with volatile organic chlorides (VOCl) [14,15,16]. As an example, degradation of different halogenated aliphatic hydrocarbons with NZVI was carried out [17] and the degradation mechanism of tetrachloroethylene (PCE), trichloroethylene (TCE), *cis*-dichloroethylene (*cis*-DCE), and *trans*-dichloroethylene (*trans*-DCE) was reported by Arnold et al. [18]. Nitrate concentration is also reduced in presence of bare NZVI [19].

Zero-valent iron is inexpensive, non-toxic and a moderate reducing reagent (standard reduction potential *E*^0^ = −0.44 V). In presence of oxygen dissolved in water, zero-valent iron is capable to oxidize organic pollutants. In a first reaction, ZVI reacts with O_2_ to produce H_2_O_2_ (Equation (1)). Consequently, formed hydrogen peroxide is reduced to water by ZVI (Equation (2)) or can react Fe^2+^, Fenton reaction, producing (hydroxyl radicals (·OH) (Equation (3)). It is important to notice that this last reaction, Equation (3), could degrade a considerable amount of organic contaminants due to its strong oxidizing capability.

Fe^0^ + O_2_ + 2H^+^ → Fe^2+^ + H_2_O_2_ (1)

Fe^0^ + H_2_O_2_ + 2H^+^ → Fe^2+^ + 2H_2_O (2)

Fe^2+^ + H_2_O_2_ → Fe^3+^ + ·OH + OH^−^ (3)

Initially, granular iron was used, mainly as a permeable reactive barrier (PRB) for chlorinated hydrocarbons, metals and metalloids (arsenic, chromium, uranium, etc.) [20,21], nitroaromatics [22] or perchlorates, among others [17,23,24,25,26,27]. However, the greater specific surface area of zero-valent nanoparticles has encouraged their use, as compared to conventional iron powder or iron filings [28,29]. Zero-valent iron has been successfully used for soil and groundwater remediation, being the PRBs developed with ZVI effective systems to limit the migration of contaminants. However, this method present several limitations since it is restricted by construction limitations of PRBs and it is not capable to target contaminant source zone [30]. In this context, many studies have shown the effectivity of nanoscale zero-valent iron (NZVI) in the last decades.

## 3. Zero-Valent Iron Nanoparticles and Nanoremediation

Zero-valent iron nanoparticles (NZVI) are more effective than macroscale ZVI, iron powder or iron filings, under similar environmental conditions [31,32,33]. Indeed, considering the exponential relationship between the specific surface area and radius of a nanoparticle, the increase on the particle size compared to microparticles increases the surface per gram several orders of magnitude [34]. The properties of the NZVIs that provide them with a great attraction for use in remediation in situ are their great reactivity towards the different families of pollutants. The reactivity of zero-valent iron is based on its ability to oxidize to ferrous or ferric iron that provides electrons available to reduce other compounds that, through the Fenton reaction, produce strong oxidants capable of reacting with contaminants making them harmless [35]. This allows addressing the decontamination in most of heterogeneously contaminated sites. The nanometric size improves the mobility through the porous medium and the low toxicity of NZVI increasing the remediation process while preserves the characteristics of the soil, so that the subsequent application of other processes such as bioremediation that can complement the treatment is not compromised. In addition, it must be noticed that the few full-scale tests have resulted in a successful remediation of main organic pollutants. This remediation technology involves a series of steps for NZVI [30] based in the transport of the nanoparticles to the area (usually in aqueous phase), and reaction with the target contaminant to form less toxic or less mobile products.

In the last years and decades, the development of nanosized materials has facilitated the application of remediation technologies based on highly efficient and versatile nanomaterials [36,37,38,39]. Among the possible nanoparticulate systems successfully used on a laboratory scale for soil decontamination, zero-valent iron nanoparticles (NZVI) have achieved very interesting and promising results (Table 1).

In fact, many studies have already corroborated the efficiency of NZVI for the remediation of contaminated groundwater and soil [29,66,67]. Moreover, nanoremediation by using zero-valent iron is the most common used method for soil and groundwater remediation both in Europe and in the United States [68]. The enhanced reactivity of the NZVIs and their high mobility allow the performance of in situ treatments through the injection of nanoparticles. These results suggest highly advantageous method for pollution remediation since their application does not specifically involve previous excavation of the soil or pumping of the groundwater [69,70]. Nanoremediation treatment commonly starts with the application of highly concentrated NZVI slurries by injection at or near the contaminated area. NZVI should be applied or attach to soils in the contaminated zone and react with the target contaminants to form less toxic or less mobile products [30].

**Figure 1:**
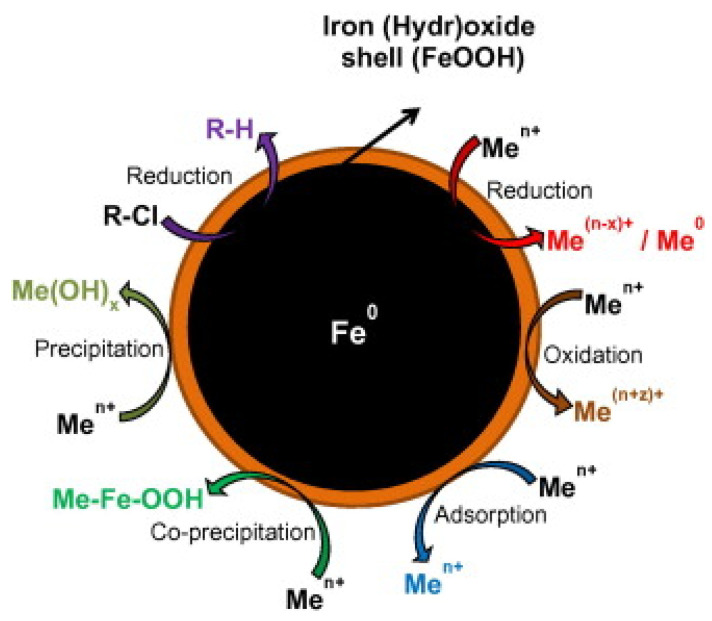
Summary of the degradation mechanism for chlorinated contaminant and metal removal by using NZVI. Reproduced with permission from [30]. Copyright 2013 Elsevier.

As an example, degradation of different halogenated aliphatic hydrocarbons with NZVI was carried out [17] and the degradation mechanism of tetrachloroethylene (PCE), trichloroethylene (TCE), *cis-*dichloroethylene (*cis-*DCE), and *trans-*dichloroethylene (*trans-*DCE) was reported by Arnold et al. [18]. Nitrate concentration is also reduced in presence of bare NZVI [19]. Figure 1 summarized various possible mechanism for the degradation of chlorinated pollutants and metals.

**Figure 2:**
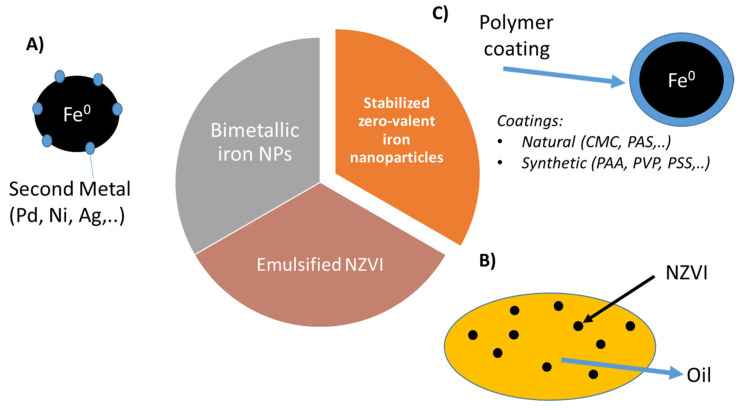
Main NZVI groups used for environmental applications: (**A**) Bimetallic iron nanoparticles, (**B**) emulsified NZVI and (**C**) stabilized NZVI.

The strong attractive forces between NZVI, mainly magnetic interactions could induce the agglomeration of the nanoparticles forming micro sized aggregates, this could reduce mobility and therefore the effectiveness of the treatment [71,72]. This low colloidal stability is even worst under environmental condition reducing significantly their applicability [73]. In order to overcome these limitations and enhance their in situ performance new types of NVZI systems have been developed. Nowadays, zero-valent iron nanoparticles used for soil and groundwater remediation can be classified in three main groups (Figure 2): (A) Bimetallic iron-based nanoparticles (BNP), (B) emulsified iron nanoparticles (EZVI) and (C) polymer coated NZVI, in which the polymer increases suspension stability and particle mobility [74].

### 3.1. Bimetallic Iron-Based Nanoparticles

The presence of two different metals in the BNPs induces a synergic effect that gives favorable properties to improve the degradation of different contaminants (Table 2). Bimetallic particles are composed of iron (or zinc) and a noble metal such as palladium (Pd), platinum (Pt), nickel (Ni), silver (Ag), or copper (Cu), being the main role of these noble metals the catalysis of the reduction reaction, thus facilitating contaminant degradation. The facile access to palladium BNPs since they are commercial have spread their use among the BNPs developed. The use of BNPs for the remediation of common halogenated contaminants and metals has been demonstrated.

**Figure 3:**
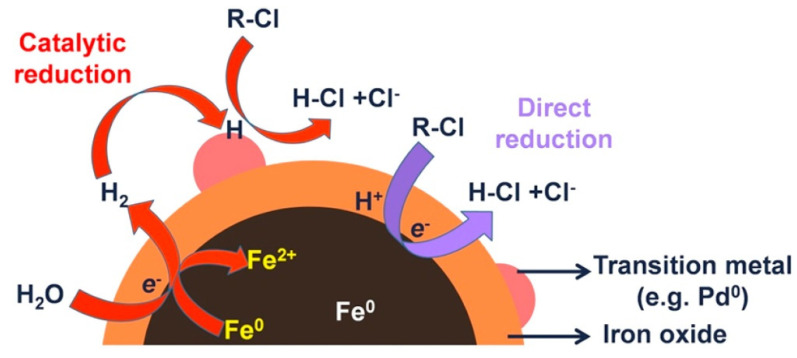
General dechlorination mechanism for BNP and NZVI. Reproduced with permission from Zhao et al. [29]. Copyright 2016 Elsevier.

Several laboratory studies have been devoted to the study of the degradation of halogenated organic compounds by using Fe/Pd bimetallic nanoparticles [28,30,90]. A scheme of a general degradation mechanism of BNP compared with NZVI it could be observed in Figure 3. Laboratory studies have shown that Fe/Pd BNPs can increase TCE degradation rates by 10−100 times [28,91]. In addition, Zhang and co-workers reported an enhanced reactivity for nanoparticles compared to microparticles [78,92,93,94]. The remediation of other chlorinated aliphatics (PCE, VC, …) has also been reported 9. Three types of chlorobenzenes (monochlorobenzene (MCB), dichlorobenzenes (DCBs), and 1,2,4-trichlorobenzene (124TCB)) have been degradated by using Fe/Pd BNPs. All tested pollutants were completely reduced by the BNPs [75]. Polybrominated diphenyl ethers (PBDEs) are commonly used as additives flame retardants in many electronic products, and even if these pollutant now is widespread, it could be specially found in e-waste recycling sites. Wang et al. evaluated the effectiveness of Fe/Pd BNPs in debromination of 2,2′,4,4′-tetrabromodiphenyl ether (BDE-47) [80]. They report a change in the debromination step due to the presence of Pd compared to the mechanism described for NZVI, this change could be responsible of the enhanced degradation of these BNPs. Similarly to brominated compounds, the dehalogenation of polychlorinated biphenyls (PCBs) were also studied by several authors [76,77].

**Figure 4:**
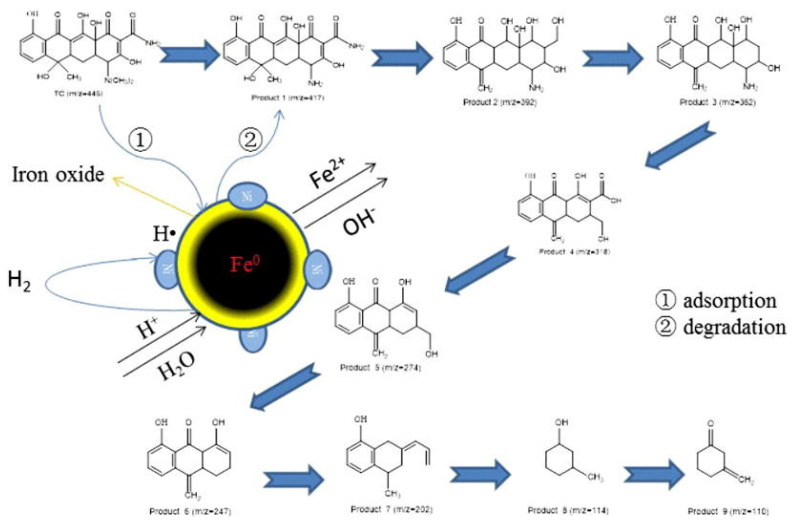
Degradation mechanism of tetracycline in presence of Fe/Ni bimetallic nanoparticles. Reproduced with permission from Dong et al. [89]. Copyright (2018) Elsevier.

Bimetallic Fe/Ni nanoparticles had been reported for the successful degradation of decabromodiphenyl ether (BDE209) [81]. The study reported a BDE209 removal efficiency up to 72% in soil for nanoparticle dosage of 0.03 g/g (at RT and pH 5.6). The degradation of trichloroethylene (TCE) with this kind of BNPs in water has been also studied. It is important to notice that the reduction by Fe/Ni was 50–80 times faster than when using nanoiron or iron fillers [79]. In addition, Fe/Ni nanoparticles were also used to degrade dyes [84,85,86]. Lin and co-workers reported the degradation of Scarlet 4BS with Fe/Ni BNP, proposing a degradation mechanism in which iron is oxidized producing hydrogen that is adsorbed in Ni surface. Around 71.2% of this dye was removed due to bimetallic nanoparticles [95]. Similarly, study of an azo dye, Orange G, degradation in presence of bimetallic Fe/Ni was demonstrated. In this study, Bokare et al. [96] described an increase in the dye degradation rate when using bimetallic nanoparticles compared to both, commercially available, micro-scale Fe powder and iron nanoparticles. Recently, an example of antibiotic removal by using Fe/Ni BNP was reported, in which a higher reduction of tetracycline concentration in water due to the treatment with Fe/Ni BNP was described compared to NZVI, the degradation process proposed by Dong et al. [89] is summarized in Figure 4.

Other zero-valent metals have also been studied for the development of BNPs, Fe/Cu bimetallic nanoparticles were used for Cr(VI) removal from soil, being the removal rate of Cr(VI) exceeded 99% in 10 min [45]. In addition, Fe/Ag BNPs have been used for debrominate two PBDEs, decabromodiphenyl ether (BDE-209) and 2,2′,4,4′-tetrabromodiphenyl ether (BDE-47). Approximately 97% of BDE-209 and 78% of BDE-47 were degrade in presence of these BNPs assisted with microwaves [97].

#### Pilot and Full-Scale Test for Bimetallic Iron-Based Nanoparticles

**Figure 5:**
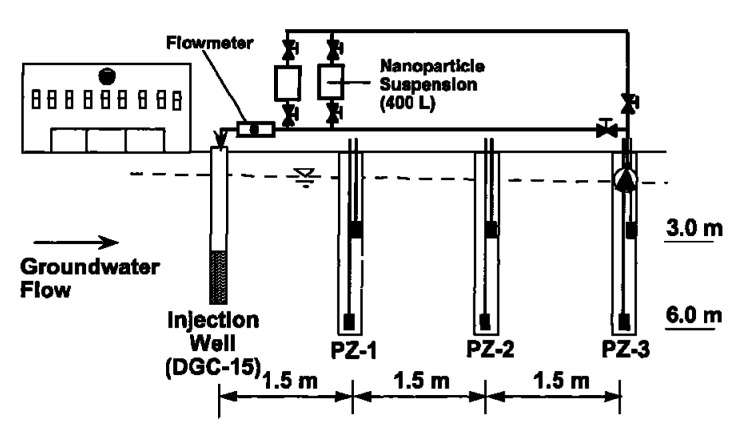
Scheme of the injection set-up. Reprinted with permission from Elliot et al. [99] Copyright (2001) American Chemical Society.

Despite the fact that most of the investigations have been carried out at the laboratory level, a few studies have indeed been performed at pilot scale or under real field conditions [98]. Elliot et al. carried out a field study by gravity-fed of bimetallic Fe/Pd nanoparticles into the contaminated groundwater [99]. Although the study presents a certain removal of TCE, the authors also reported the necessity of using higher nanoparticle concentrations and more frequent dosing pattern, in order to achieve more relevant contaminant reduction. In Figure 5, the scheme of the set-up developed by Elliot and co-worked could be observed, in which an injection point flow by three piezometers was used.

Though several studies have reported the use of bimetallic nanoparticles for aquifer and groundwater remediation, there are only a few studies devoted to soil remediation (Table 3). As an example, a study on the application of BNPs for soil and groundwater remediation was performed on three US Navy facilities in which the concentrations of PCE, TCE, and other secondary pollutants were successfully decreased. The treatment with BNP provided a decrease in the total average concentration of volatile organic compounds (VOC) of 74 percent, after six months of tests [98].

However, these bimetallic nanoparticles present several disadvantages such as a short lifetime due to surface passivation [30,79], structural changes [75], and an environmental risk associated to the secondary metal, especially for Fe/Ni BNPs, due to the toxicity of the obtained by-products and reactivity decrease if they are not eliminated [79]. In this context, the selection of the less potentially hazardous metal is highly important for this remediation, so metals such as nickel should be avoided. On the other hand, the passivation of the BNP could be reduced with polymer coating that protect them until they reach the contaminants and, additionally, it could improve their transport through the soil [100].

### 3.2. Emulsified Zero-Valent Iron

Another iron nanoparticle-based product to be highlighted for environmental remediation is emulsified zero-valent iron (EZVI) [103]. The aim of this kind of systems is to deliver NZVI in an oil–water emulsion, which eases the transportation into the contaminated zones and reduces the NZVI’s degradation [104]. EZVI is a surfactant-stabilized, biodegradable emulsion that forms emulsion droplets consisting of an oil–liquid membrane surrounding zero-valent iron (ZVI) particles in water. These emulsions are able to degrade chlorinated hydrocarbons [105]. EZVI can be fabricated from ZVI (macroscopic) to microscale or nanoscale, or as a combination of both. The use of formulations in which micro- and nanoparticles are combined reduces the cost of the materials without losing the benefits provided by the nanoscale iron, since the microparticles are less expensive [74]. The outer oil membranes are hydrophobic, making them similar to some common contaminants such as DNAPL (dense, non-aqueous liquid phase) or TCE, so that the EZVI droplets are miscible with these contaminants. When the emulsion drops are in close contact with TCE, they are mixed and then, TCE is diffused inside the droplet where, in contact with the zero-valent iron, is degraded. A concentration gradient is established due to the diffusion of TCE inside the drop and the subsequent migration of the reaction by-products to the surrounding aqueous phase, thus improving the degradation process [105]. In addition, some studies report that the use of vegetal oil for this kind of emulsions can improve biodegradation processes [106,107].

EZVI could be considered an environmental friendlier approach compared to the bare NZVI and BNP. The encapsulation of the NZVI on biodegradable oil improve the mixture of EZVI with DNAPLs reaching the organic pollutants on groundwater or water flows that could be difficult to access with other technologies [108].

#### Pilot and Full-Scale Test for Emulsified Zero-Valent Iron

EZVI has been used to clean up contaminated soil and groundwater in several locations (Table 4). In a field experiment performed at Parrick Island (SC, USA), PCE and TCE concentrations were reduced by the application of EZVI using two different delivery methods: pneumatic injection and direct injection. A significant decrease in groundwater PCE (>85%) and TCE (>85%) concentrations was reported. However, the authors expressed their concern about the efficiency of these methods, since they detected uncertainties in the estimations due to a possible mobilization of DNAPL during and after the EZVI injection process [109]. Often, a compromise between the advantages and disadvantages of the remediation technology is required. For example, the excess on the contaminant mobility induced by EZVI could be reduced with the optimization of the emulsion components, usually surfactants, or complementarily pumping out the mobilized DNAPL if the site and the required technology are compatible.

In addition, comparing EZVI technology with the BNP, the first one presents several advantages refereed to the cost and the homogeneity of the reagents. The best of our knowledge the fabrication of the BNPs still very limited due to the cost and synthetic limitations that prevent them of being massively fabricated.

**Figure 6:**
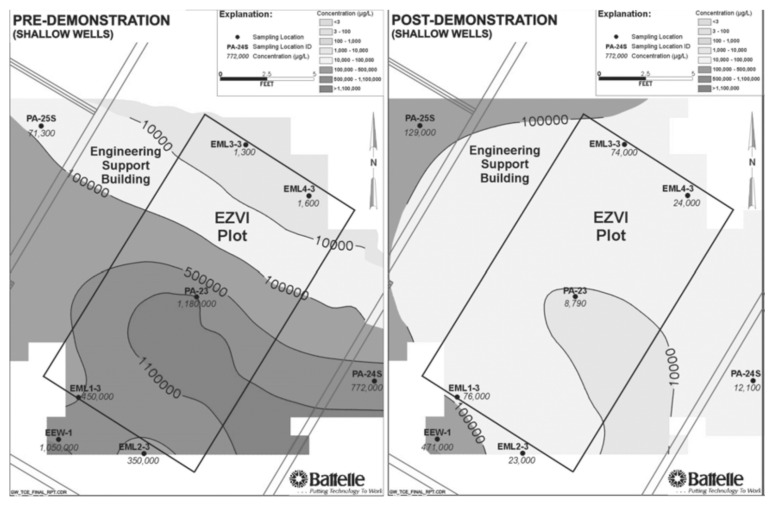
Scheme of the injection set-up. Reprinted with permission from Geiger et al. [105]. Copyright (2006) Wiley.

In a soil and groundwater remediation initiative developed at Cape Canaveral Air Force Station (FL, USA), O’Hara and co-workers reported and effective contaminant reductions when ENZVI was applied to DNAPL. The concentration in TCE in soil was reduced to more than 80%, whereas TCE concentration in the groundwater was reduced by 60% [105]. In Figure 6, the concentration contours of TCE in groundwater of shallow wells could be observed in the pre- and post-demonstration carried out on Cape Canaveral Air Force Station, Florida, showing reported reduction. Similarly, in a field test performed in an industrial site at Patrick Air Force Base (FL, USA), an initial TCE concentration of 150,000 μg/L was reduced to 3580 μg/L by a treatment with EZVI introduced by high pressure pneumatic injection [110].

### 3.3. Polymer Coated NZVI Particles

Nanoparticles present a high reactivity due to their large surface area, being this characteristic crucial for the rapid degradation of contaminants, compared to zero-valent iron or microparticles [74]. In spite of their effectiveness as a decontaminant agent, NZVIs present some weaknesses including a lack of stability, their rapid passivation and limited mobility, since the nanoparticles tend to aggregate rapidly in water solution. In addition, zero-valent iron has a high affinity for oxygen. This tendency to oxidize rapidly causes a passivation of the nanoparticles in contact with the air or in aqueous medium [73]. In order to reduce these problems, different polymer coatings have been used as a strategy to protect the nanoparticles against oxidation and promote a greater degree of dispersion. Indeed, polymer-stabilized nanoparticles present a higher stability in aqueous suspension and a better soil transportability than non-coated NZVIs [29,112]. Nanoparticle stabilization increases the remediation capability of the NZVIs.

**Figure 7:**
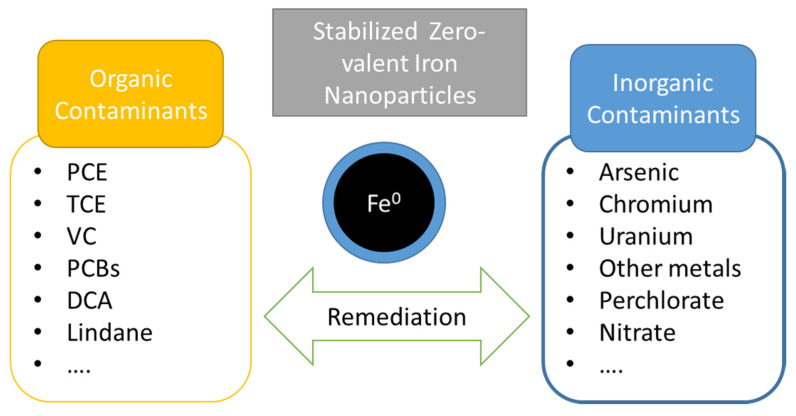
Summary of the main contaminants remediated with stabilized NZVI.

Since the polymer coating or stabilizer are in charge of enhance the colloidal stability of NZVI, their adequate selection is also an extremely important factor. The intrinsic properties of the polymers must be considered being the biocompatibility and/or biodegradability extremely important in order to do no increase the environmental problems. In addition, the presence of polymers, mainly of biopolymers, could enhance the biodegradation since they serve as an additional nutrient source for microorganism [113]. In recent years, biodegradable coatings have been incorporated to NZVI surfaces in order to improve the dispersion of the nanoparticles, increase their stability, and protect the reactive centers until contact with the target contaminant. A great effort has been made to develop effective polymer coatings to maximize the remediation capability of NZVI. In general, the higher surface reactivity and the strong interparticle interaction of bare NZVIs make their coating difficult since not many polymer could meet the specific requirement to guarantee their good dispersability, compared to other nanoparticles. Various surfactants and polymers have been successfully used as stabilizers of NZVIs and, according to the remediation results, coating with polymers, both natural and synthetics, lead to improved remediation results [29]. Polymer coatings not only prevent the aggregation of the nanoparticles, but also, in some cases, they can also serve as a food or energy source for microorganisms involved in bioremediation processes. NZVI coating has been developed by using synthetic polymers such as poly (N-vinylpyrrolidone) (PVP), polystyrene sulfonate (PSS), polyacrylic acid (PAA) and its derivatives, or carboxymethycellulose (CMC), among others (Table 5). In addition, some biopolymers have been used as nanoparticle coatings such as starch, Xanthan gum or guar gum [114,115,116]. Polymer-stabilized zero-valent nanoparticles have been extensively studied for the remediation of both organic and inorganic pollutants (Figure 7). Even more, several studies have reported a successful remediation of chlorinated hydrocarbons (TCBs, PCE, TCE, DCE, VC, DCA, and lindane) and inorganic contaminants in soil and groundwater [117,118].

#### 3.3.1. NZVI Coated with Synthetic Polymers

Many formulations of synthetic polymers have been used for coating NVZI, being the most of them polyelectrolytes and few neutral polymers. Negatively charged polyelectrolytes are used since they are capable to form a polyelectrolyte layer that induce strong electrostatic repulsions [124,131]. Poly (acrylic acid), polystyrenesulfonate, polyoxyethylene sorbitan monolaurate, polymethacrylic acid, and di-/triblock copolymers have been used as NZVI coatings and tested against different pollutants. TCE [66] and lidane [64] have been degraded with PAA-coated nanoparticles. The use of other anionic polyelectrolytes such as polystyrenesulfonate (PSS) significantly decreases the aggregation degree and, in consequence, improves the diffusion of the particles through the medium [125,132]. Sirk et al. studied the effect of the coating with different block copolymers based on poly (methacrylic acid) (PMMA), poly (methyl methacrylate) or PSS, among others [124]. From their test in a soil model, they concluded that the electrostatic repulsion between the polyelectrolyte-coated NZVI and the negatively charged soil surfaces reduce the adhesion and therefore enhanced the mobility of the nanoparticles through the soil. Similarly, triblock copolymers had been studied as NZVI coatings. Saleh and co-workers analyzed the effect of amphiphilic triblock copolymer coating PMAA-b-PMMA-b-PSS [126]. The polymer layer was physisorbed on the nanoparticles’ surface and promoted the colloidal stability of the NVZIs. The evaluation of these nanoparticles on a model soil indicated good mobility. Moreover, they can be absorbed in oil–water interface improving their capacity to reach chlorinated pollutants in order to degrade them. In another example of triblock copolymers, polyvinyl alcohol-co-vinyl acetate-co-itaconic acid (PV3A) copolymer was used as a nontoxic and biodegradable coatings of NZVI [119]. This coating improved several properties such as surface chemistry and particle stability, and therefore NVZI’s mobility through the soil. In addition, the study demonstrates an effective removal of TCE. Finally, it is important to notice that no sedimentation of these nanoparticles were observed for over 6 months.

In addition to polyelectrolytes, neutral synthetic polymer have been used for the fabrication of stabilized NZVIs. For example, neutral polyethylen glycol (PEG) and polytetrahydrofuran (PTHF) have been studied for lindane degradation [65]. Cirtiu et al. comparatively studied colloidal stability of CMC, PAA, PSS, and polyacrylamide (PAM) [123]. The stability of CMC and PAA, both polyelectrolytes with carboxylic functionalities, present very similar stability, being the more stable formulations. PSS present similar stability than PAM, neutral synthetic polymer, which at the same time both of them are 13 times more stable than non-coated NZVI. Among the neutral polymers, polyvinylpyrrolidone (PVP) is the most commonly used synthetic polymer. Several studies indicated good colloidal stability and successful decontamination effect of PVP-coated NZVIs on the removal of TCE and tetracycline (TC), being the dechlorination efficiency for TCE around 85% [120,121,133]. However, Sakulchaincharoen and co-workers described a lower performance of PVP-coated nanoparticles compared to CMC-coated NZVI in TCE degradation rate, however, when the ratio is normalized to the surface area PVP-coated NZVI presents a higher rate [122].

#### 3.3.2. NZVI Coated with Natural Polymers

Natural polymer used as NZVI coating could be classified according to the driving effect that induces the stabilization as polyelectrolytes and viscosity modifiers. For example, CMC is adsorbed to the nanoparticle forming a negatively charged layer that promotes electrostatic repulsions with the surrounding nanoparticles. These could be used in a porous medium, manipulating their range by modifying the pressure and flow of the injection [134,135]. A comparative study of NZVI coated with PSS, polyaspartate (PAS) and CMC was reported by Kim et al. [125]. All the formulations present high stability, being the coating layer disrupted only after 4 months. It is important to notice that the mobility through sand columns of the stabilized nanoparticles after 4 months remains the same as the freshly prepared ones. Similarly, lindane was treated in solution using NZVI coated with PAS and CMC. A complete elimination of lindane in 72 h was reported for all studied coatings [64,65]. Considering the influence on the stability of the CMC and the nanoparticle size, He and co-workers reported CMC coating capable to adapt their nanoparticle size and dispersability as a function of several synthetic variations [100]. This adaptation could significantly improve the applicability of these CMC-coated nanoparticles since they can be adapted to the diversity of conditions in different soils and/or groundwater. Overall, the synthesized formulations present better stability and a 17 fold higher degradation rate than bare-NZVI.

A part form natural polyelectrolytes, among the natural polymers used for NZVI stabilization there are some of them that they can stabilize NZVI’s slurry by increasing the viscosity. The viscosity increase of nanoparticle slurry reduced the aggregation and sedimentation of NZVI trapped on it. Comba et al. reported a xanthan gum (XG) stabilized NZVI that maintains its stability for more than 10 days [130]. XG formulations of this study are stable to ionic strengths variation in a range between 6 × 10^−3^ and 12 mM. Similarly, a good stability was observed by Tiraferri et al. for guar gum (GG) gels, their aggregation and sedimentation were reduced and they were stable to a high ionic strength media [136]. In addition, Xue and co-workers studied this kind of stabilization on zero-valent iron nano- and microparticles by using XG and GG formulations and a mixture of both [116]. In their study, formulations obtained by XG or GG present a good stability against the aggregation and sedimentation for few hour. However, when these two biopolymers are mixed the resulting materials present an improved stability of over a day due to the interactions between them.

#### 3.3.3. Pilot and Full-Scale Test for Polymer Coated NZVI Particles

Some pilot and full-scale tests have carried out by using stabilized NZVI (Table 6). In Hamilton Township, New Jersey (USA), a remediation strategy based on this nanotechnology showed positive results. The NZVI were injected in two phases and the duration of the test was 30 days. The results showed a decrease in the concentration of chlorinated contaminants of up to 90 percent [32]. There are a large number of new trials at pilot and field scale; some of the most recent are in progress or the results are not known yet. The contaminants most frequently treated by these methods are chlorinated solvents such as TCE, PCE, TCA, and VC. Most of the pilot and full-scale tests have been carried out in USA: for example, soil remediation through the application of NZVI was conducted in the Naval Air Station of Jacksonville (USA) and the Naval Air Engineering Station of Lakehurst (USA). Both areas presented high levels of TCA, DCE, TCE, and PCE. After the trial, contamination levels decreased by 80–90% [98].

In Europe, only few full-scale tests have been carried out. In 2007, NZVIs stabilized with poly(acrylic acid) were tested in Bornheim (Germany) to remediate PCE from the aerospace industry. The contamination was spread over an area of several square kilometers, down to a depth of 20 m, and the efficiency of the remediation process was 90% [137]. In addition, 2 years after the application of NZVI, a further reduction in contaminant concentrations was observed. Another test developed in the EU was carried out in the Czech Republic (Horice and Pisecna). In two contaminated areas (7 and 2 km^2^), 82 injection wells were constructed and 300 kg of NZVI were injected. The results revealed a contamination reduction of 60–75 and 90% for Horice and Pisecna, respectively [137].

It is important to notice that, commonly, more than 100kg are used on the remediation of full-scale area of around 2 km^2^, considering NZVI power ranged $66 to $88/kg [137], the use of stabilized NZVI that present less passivation being more efficient, is a highly attractive alternative. Zhao et al. [29] estimated that starch-stabilized and CMC-stabilized nanoparticles cost ranges from $100 to 120/kg, so considered their hypothetical cost and the increase of the active nanoparticles reaching the contaminant due to their higher stability, it could be considered that this kind of NZVI as an interesting alternative for the soil remediation. In addition, those nanoparticles coated with natural polymers, do not added any potential risk by-product since they are biodegradable

## 4. Limitations and Risks Derived from NZVI Nanoremediation

Even if many studies have demonstrated the success of NZVI-based nanoremediation, there are still many uncertainties regarding this technology that must be addressed, such as the possible formation of microsized cluster due to the nanoparticle aggregation, the mobility lack of bare NVZI or the potential ecological or human risk derived from the used of nanoparticles [138,139,140]. Among the potential environmental risk, their nanosize could negatively affect the drinking water if the NZVI are uncontrollably transported and, also, there are some important lack on studies on the ecotoxicity or bioaccumulation on pilot and full-scale remediation.

Until now, it could be considered that there is an important lack of information on the long-term effect on the environment of nanoparticulate systems, in general, and concretely in NZVI. Some studies reported a concern on its toxicity for many living organism [141,142,143]; however, there are also many studies that reported no significant influence on microbial community [129,144,145]. In addition, Saccà et al. [146] reported that the toxicity of NZVI on soil organisms was higher in vitro assays performed in lab that in soil. In this context, it is clear that more systematic studies must be carried out to evaluate the possible application risks on this technology.

In the light of these risks and the insufficient understanding of some of the derived hazards, many studies and a clear legislation on this are required. The standardization of the testing methods, transportation, and remediation could significantly reduce some of these lacks [141]. Five points could be considered in future studies in order to reduce these limitations: (1) Improve in the management considering the recovery of NZVI when is possible; (2) evaluations the effects of the NZVI in the living organism considering that they could, in some point, enter in the food chain, so not only on human and wildlife health should be considered, also on lower microorganisms; (3) analysis of the NZVI aging effects and the risks contaminant degradation by-products on the ecotoxicity; (4) the soil nature must be considered in addition to the contaminants present in it; (5) in some cases, it could be considered the combination of nanoremediation with other remediation technologies that improve the biological aspect of the soil, such as bioremediation of phytoremediation.

## 5. Conclusions

The environmental remediation by NZVI technology has been widely tested at the laboratory level against a large amount of contaminants, offering very promising results in order to solve heterogeneous pollution problems in the same intervention. However, as it has been previously mentioned, there are still many uncertainties regarding NVZVI remediation.

In order to overcome or reduce these problems and uncertainties, the stabilized NZVIs, mainly polymer-coated NZVIs, have the benefit of offering great versatility in terms of its use in locations with different characteristics and different types of contaminants due to their improved colloidal properties compared to bare-NZVI. Each contaminated site requires a specific design to address its decontamination; however, they are remarkable the results obtained for CMC-coated nanoparticles since these nanoparticles present an excellent mobility, a degradation ratio and the CMC could be easily biodegraded due to its polysaccharide nature. In this context, the coating based on greener polymers, natural and biodegradable, which are environmentally friendly with the water and soil ecosystems, but at the same time could improve the transportation and the colloidal stability of the nanoparticles seems be to the future of this technology.

## Figures and Tables

**Table 1:** Summary of pollutants remediated by zero-valent iron nanoparticles (NZVI)

Pollutants	NZVI Based Treatment	Reference
Heavy metals and metalloids		
As	NZVI	[40,41,42]
Pb + Zn	NZVI	[43]
Pb	NZVI + citric acid	[44]
Cr(VI)	NZVI/Cu; NZVI/biochar; starch stabilized NZVI	[45,46,47,48]
Pb + Cd + Cr	NZVI + activated carbon	[48]
Sb(V)	NZVI + humic acid coated; NZVI	[49]
U	NZVI	[50]
Hg	NZVI	[51]
Ni	NZVI	[52]
Ag	NZVI	[52]
Cd	NZVI	[51]
Other inorganic compounds		
Perchlorate	NZVI	[51]
Nitrate	NZVI	[51]
Organic compounds (pesticides, polychlorinated hydrocarbons, chlorobenzenes, colouring agents, etc.)		
PAHs	NZVI; NZVI + SDS	[42,53,54,55]
TCE	CMC-NZVI	[56]
TCE + PCB	Starch-stabilized Fe/Pd	[57]
Ibuprofen	NZVI	[58]
DDT	NZVI	[59,60]
TNT	NZVI	[61]
2,3,7,8-TCDD	NZVI + Tween 80	[62]
TCC	NZVI	[51]
Bromoform	NZVI	[51]
Chloroform	NZVI	[51]
Dibromochloromethane	NZVI	[51]
Dichloromethane	NZVI	[51]
Dichlorobromomethane	NZVI	[51]
Chloromethane	NZVI	[51]
*N*-nitrosodimethylamine	NZVI	[52]
Hexachlorobencene	NZVI	[52]
PCB	NZVI + saponin	[63]
DDT	NZVI	[52]
Lindane	NZVI	[64,65]
Pentachlorophenol	NZVI	[52]
Chrysoidin	NZVI	[51]
Tropaeolin O	NZVI	[51]

**Table 2:** Summary of the main pollutants treated with bimetallic iron-based nanoparticles (BNPs).

Components	Contaminant	Reference
Fe/Pd	Chlorobenzenes	[75]
Fe/Pd	Polychlorinated biphenyls (PCB)	[76,77]
Fe/Pd and Fe/Ni	Chlorinated aliphatics (PCE, TCE, VC, …)	[28,59,78,79]
Fe/Pd and Fe/Ni	Polybrominated diphenyl ethers (PBDEs)	[80,81]
Fe/Cu, Fe/Al and Fe/Ni	Chromium, Copper	[45,82,83]
Fe/Pd, Fe/Zn and Fe/Ni	Dyes (Orange G, Congo red, Orange II, …)	[84,85,86,87,88]
Fe/Ni	Tetracycline	[89]
Fe/Ni	Triclosan	[82]

**Table 3:** Summary of pilot and field scale studies of BNPs.

Pollutants	Concentration Decrease	Addition Method	Site	Comments	Time for Study	Reference
TCE	96%	Gravity-fed	Groundwater	High dosage of BNPs needed	n/a	[99]
PCE, TCE; Others	n/a	n/a	Soil and groundwater	n/a	n/a	[98]
VOCs	74%	n/a	n/a	n/a	Six months	[98]
VOCs (ethane, TCE, TCA, DCE, DCA)	65–96%	Gravity-fed and recirculate	Groundwater	Pd/Fe BNPs	1 year	[101]
VC	50–99%	Injection	Groundwater	Pd/Fe BNPs	Six months	[102]

**Table 4:** Summary of pilot and full scale tests for emulsified zero-valent iron (EZVI).

Pollutants	Concentration Decrease	Addition Method	Site	Comments	Location	Reference
TCE, TCA, DCE, DCA	TCE and TCA > 65–96%	Gravity-fed and recirculate	groundwater	Polymer coated nanoparticles	Naval Air Station Jacksonville (FL, USA)	[101]
PCE, TCE	PCE > 85% TCE > 85%	Pneumatic injection, direct injection	groundwater	Uncertainties in the estimations	Parrick Island (SC, USA)	[109]
TCE	TCE > 80 (DNAPL) TCE > 60% (groundwater)	n/a	DNAPL, groundwater	n/a	Cape Canaveral Air Force Station (FL, USA)	[105]
TCE	TCE > 95%	n/a	n/a	n/a	Patrick Air Force Base (FL, USA)	[110]
Chlorinated VOCs	>86%	Pneumatic injection	Soil and groundwater	2.5 years monitoring	Marine Corps Recruit Depo. Parris Island (SC, USA)	[111]

**Table 5:** Summary of main examples of polymer coated NZVI.

Coated Polymer	Improvement	Contaminant	Reference
Synthetic Polymers			
PAA	Transportability	TCE, Lindane	[64,66]
PV3A	Stability	TCE	[119]
PEG	Stability	Lindane	[65]
PTHF	Stability	Lindane	[65]
PVP	Stability	TC, TCE	[120,121,122]
PSS	Stability	n/a	[123,124,125]
PAM	Stability	n/a	[123]
PMAA-b-PMMA-b-PSS	Stability	TCE	[124,126]
OMA	Transportability	n/a	[127]
Natural Polymers			
CMC	Stability, Transportability	TCE, PCB, Lindane, Cr(VI)	[64,100,122,125,128,129]
PAS	Stability	Lindane	[64,125]
XG	Stability, Transportability	n/a	[116,130]
GG	Stability, Reactivity	TCE	[116,122]

**Table 6:** Summary of pilot and full-scale tests for polymer coated NZVI particles.

Pollutants	Conc. Decrease	Addition Method	Site	Comments	Location	Reference
Chlorinated compounds	>90%	Injection in two phases		30 days	Hamilton Township, New Jersey (USA)	[32]
TCA, DCE, TCE, PCE	80–90%	n/a	Soil	n/a	Naval Air Engineering Station of Lakehurst (USA)	[98]
TCA, DCE, TCE, PCE	80–90%	n/a	Soil	n/a	Naval Air Station of Jacksonville (USA)	[98]
PCE	90%	n/a	Soil	2 years after, more reduction	Bornheim, Germany (Europe)	[137]
PCE, TCE, DCE	60–75% for Horice and 90% for Pisecna	Injection (82 injection wells)	Soil	n/a	Czech Republic (Horice and Pisecna)	[137]
Chlorinated compounds	>90%	n/a	n/a	30 days	Hamilton Township, New Jersey (USA)	[32]

## Abbreviation

·OH	hydroxyl radical
124TCB	1,2,4-trichlorobenzene
2,3,7,8-TCDD	2,3,7,8-tetraclorodibenzo-p-dioxine
BDE-209	decabromodiphenyl ether
BDE-47	2,2′,4,4′-tetrabromodiphenyl ether
BMP	bimetallic iron-based nanoparticle
*cis*-DCE	*cis*-dichloroethylene
CMC	carboxymethylcellulose
DDT	Dichlorodiphenyltrichloroethan
DNAPL	dense non-aqueous liquid phase
EZVI	emulsified iron nanoparticles
GG	guar gum
MCB	monochlorobenzene
n/a	Not available
NZVI	Nanoscale zero-valent iron
OMA	Olefin maleic acid
PAA	Poly(acrylic acid)
PAH	polycyclic aromatic hydrocarbons
PAM	polyacrylamide
PAS	polyaspartate
PCB	polychlorinated biphenyl
PCE	Tetrachloroethylene or Perchloroethylene
PEG	polyethylen glycol
PMMA	poly(methacrylic acid)
PRB	permeable reactive barrier
PSS	polystyrene sulfonate
PTHF	polytetrahydrofuran
PV3A	polyvinyl alcohol-co-vinyl acetate-co-itaconic acid
PVP	polyvinylpyrrolidone
TC	tetracycline
TCA	trichloroethane
TCC	Triclocarban
TCE	trichloroethylene
TNT	2,4,6-trinitrotoluene
*trans*-DCE	*trans*-dichloroethylene
VC	vinyl chloride
VOC	Volatile organic compound
VOCl	Volatile organic chlorides
XG	xanthan gum
ZVI	Zero-valent iron
